# Teaching life cycle assessment in higher education

**DOI:** 10.1007/s11367-020-01844-3

**Published:** 2020-12-17

**Authors:** Tobias Viere, Ben Amor, Nicolas Berger, Ruba Dolfing Fanous, Rachel Horta Arduin, Regula Keller, Alexis Laurent, Philippe Loubet, Philip Strothmann, Steffi Weyand, Laurie Wright, Guido Sonnemann

**Affiliations:** 1 Institute for Industrial Ecology (INEC), Pforzheim University, Pforzheim, Germany; 2 Interdisciplinary Research Laboratory in Life Cycle Assessment and Circular Economy (LIRIDE), Université de Sherbrooke, Quebec, Canada; 3 ISM, UMR 5255, Université de Bordeaux, Talence, France; 4 PRé Sustainability B.V, Amersfoort, Netherlands; 5 Institute of Natural Resource Sciences, Zurich University of Applied Sciences, Wädenswil, Switzerland; 6 Section for Quantitative Sustainability Assessment, Technical University of Denmark (DTU), Kgs. Lyngby, Denmark; 7 Forum for Sustainability Through Life Cycle Innovation e.V. (FSLCI), Berlin, Germany; 8 Institute IWAR, Material Flow and Resource Economy, Technical University of Darmstadt, Darmstadt, Germany; 9 Solent University Southampton, Southampton, UK

**Keywords:** LCA, Life cycle thinking, Learning outcomes, Competency levels, Teaching approaches and content, Pedagogy

## Abstract

**Purpose:**

Scientific Life Cycle Assessment (LCA) literature provides some examples of LCA teaching in higher education, but not a structured overview of LCA teaching contents and related competencies. Hence this paper aims at assessing and highlighting trends in LCA learning outcomes, teaching approaches and developed content used to equip graduates for their future professional practices in sustainability.

**Methods:**

Based on a literature review on teaching LCA in higher education and a collaborative consensus building approach through expert group panel discussions, an overview of LCA learning and competency levels with related teaching contents and corresponding workload is developed. The levels are built on the European Credit Transfer and Accumulation System (ECTS) and Bloom’s taxonomy of learning.

**Results and discussion:**

The paper frames five LCA learning and competency levels that differ in terms of study program integration, workload, cognitive domain categories, learning outcomes, and envisioned professional skills. It furthermore provides insights into teaching approaches and content, including software use, related to these levels.

**Conclusions and recommendations:**

This paper encourages and supports higher educational bodies to implement a minimum of ‘life cycle literacy’ into students’ curriculum across various domains by increasing the availability, visibility and quality of their teaching on life cycle thinking and LCA.

## Introduction

Throughout the last decades, a shift in many societies has been seen with politicians introducing stronger regulations focusing on sustainability aspects, customers demanding more sustainable products and companies increasingly offering sustainable products (Mittelstaedt et al. 2014). Consequently, there is a need for sustainability professionals, who can guide institutions and organizations of all types and sizes through a transition process towards sustainable societies.

In this setting, universities play an important role. Around the world, they have reacted to these increasing societal needs with the development of entire sustainability programs and/or introduction of sustainability aspects into their existing curricula (Shriberg and MacDonald 2013). Along with an increased focus on sustainability, several universities have integrated the concept of life cycle thinking in their programme. Life cycle thinking and the associated life cycle assessment (LCA) methodology are increasingly taught to equip students with an ability to address complex sustainability challenges (Roure et al. 2018).

Life cycle thinking aims to increase the sustainability of a product or system along its entire value chain by reducing environmental impacts and at the same time increasing socio-economic performances (UNEP 2020a). LCA primarily focuses on environmental impacts alone (ISO 14,040/14,044 2006), assessing quantitatively the environmental impacts of products and services along their value chains. Over the past decades, methodological developments in the direction of environmental and societal Life Cycle Costing (Swarr et al. 2011), Social LCA (Benoit and Mazijn 2009) and Life Cycle Sustainability Assessment (Klöpffer 2008; Valdivia et al. 2012) have also been made, as have numerous differentiations and brandings, including carbon and water footprints (ISO 14067,2018; ISO 14046,2014) or the EU’s Product Environmental Footprint and Organisation Environmental Footprint (EC JRC 2020; Bach et al. 2018; Pant et al. 2012). In the following, the term LCA is used in a broad sense and captures these different concepts and methodologies.

According to the authors’ experience, teaching in this field usually starts with a short introduction with life cycle thinking followed by the core methodology of environmental LCA, and then conveys the developments and specializations mentioned above. Depending on their future careers, students may require different levels of understanding of LCA. For example, some might only be required to understand the concept of life cycle thinking and its importance in assessing and managing sustainability aspects. Others may require to be LCA-literate so they can understand and use LCA results for their decision-making. Others can also become fully proficient in LCA application to be able to perform full-scale LCA studies. The level of LCA competency required from students therefore varies to a high degree. Depending on the university and scientific field, the level of LCA competency that is currently taught can vary considerably (Olsen et al. 2018). While some universities only introduce the concept as (a small) part of regular courses, others offer dedicated courses, modules, minors, or majors on the subject. A systematic integration of life cycle approaches and tools in a curriculum is still rare, as outlined by Roure et al. (2018) and Cosme et al. (2019).

While there are some examples in scientific literature on how LCA is taught – which will be further explored within this paper – a more structured guidance and overview on LCA learning and competency levels, associated with matching teaching approaches and content, is not available. However, this could contribute to a better understanding and implementation of LCA teaching in higher education. Such an overview could enable a better exchange of knowledge among educators on the subject and allow students to identify available programs around the world. It would also enable prospective employers to better understand students’ qualifications and could support and encourage more universities to introduce LCA courses. It could also help curriculum developers to decide on appropriate methods and credits to provide the desired qualifications to the students.

This paper aims to fill this gap and intends to shed some light on LCA teaching in higher education by answering the research question: *What LCA learning outcomes, teaching approaches and contents can be recommended to equip graduates for their respective future professional practices?*

Initially, this question had been raised within the Forum for Sustainability through Life Cycle Innovation (FSLCI) in 2017. FSLCI was created as global network for LCA professionals in 2015 in response to and to further strengthen the global mainstreaming of LCA (FSLCI 2020). The initial question led to the foundation of a working group on LCA in higher education with the mission to encourage and support higher educational bodies to apply a minimum of literacy on life cycle thinking and LCA, shortly referred here as ‘life cycle literacy’, to students across various domains by increasing the availability, visibility and quality of their teaching on life cycle thinking and LCA.

## Methods and materials

For describing and structuring of learning outcomes and LCA qualifications at various levels and across a range of subjects from marketing, through engineering, to dedicated industrial ecology courses, first, the state of the art of studies on LCA is identified and evaluated based on a literature review. Second, a generic framework on LCA learning and competency is developed making use of Bloom’s taxonomy of learning. To showcase the use of the framework for teaching LCA in higher education, LCA teaching activities, material, and contents are categorized according to the proposed framework.

### Literature review

A literature review was performed aiming to identify the relevant scientific papers published in the past years on LCA teaching. The review did not collect all possible types of documentation and material from the numerous LCA courses taught around the world (many of them are not publicly accessible). It rather aimed at summarizing the current state of research on teaching LCA in higher education. Hence, the literature review covered databases of scientific research (JSTOR, ScienceDirect, ResearchGate) using the keywords “teaching”, “higher education” and “learning” combined with “Life Cycle Assessment”, “Life Cycle Analysis”, “LCA”, or “lifecycle” (with different spelling).

Since many of the search results also described course formats in more detail, all studies identified were assessed based on the following criteria:

• Scope: description of a teaching experience (in an university and /or country) or a generic proposal of curriculum and/or insights to develop an LCA course;
• Focus: teaching method, LCA competences, learning outcomes or literature review;
• Course content: details of the content, including topics taught;
• LCA software: use of spreadsheet, streamlined software or full LCA software;
• Number of hours and/or credits: workload of the course;
• Students background: engineering and technical sciences, or business or social sciences;
• Target audience of the course: bachelor, master or doctoral;
• General information: country and journal.

### Nominal group technique for conducting expert panels

The development of the framework and the categorization of LCA contents and activities are results of expert panels. Expert panels or stakeholder workshops are commonly used ways to discuss and develop consensus in complex subjects from medical practice to specific resource efficiency topics. In the field of LCA, expert panels have been used to build consensus on the development of guiding principles for LCA databases (e.g. Pennington et al. 2010), definition of impact assessment indicators (e.g. Frischknecht et al. 2016) or weighting factors (e.g. Pizzol et al. 2017).

In this study, expert panels were conducted based on the five-step nominal group technique as described in Potter et al. (2004) and Harvey and Holmes (2012) to build stepwise consensus on the learning outcomes and achievable competencies in higher education on LCA. FSLCI invited about 40 internationally recognized universities and academic institutions and announced open expert panel workshops at the 2017 and 2019 Life Cycle Management Conferences. Three semi-structured expert panels were conducted in Luxembourg in 2017, at the University of Bordeaux in 2019, and due to the COVID-19 crisis online in 2020. The experts, who participated in the panels represented the following stakeholders of higher education in LCA:

• Université de Bordeaux and the engineering school Bordeaux INP (France), where LCA is integrated into general and professional bachelor studies (natural sciences and engineering), in master (chemistry and engineering), and PhD programs.
• Technical University of Denmark (DTU), which is an engineering university, where sustainability assessment and life cycle thinking are introduced to all students at BSc, MSc and PhD levels (via mandatory courses), and where LCA has been comprehensively taught at MSc level for the past 20 years.
• Technische Universität Darmstadt, where the Chair of Material Flow Management and Resource Economy provides LCA teaching for bachelor (environmental and civil engineering), master (various engineering and material sciences) and PhD programs.
• Pforzheim University’s business school (Germany), where LCA is the core content of a master program provided by the Institute for Industrial Ecology and major part of several PhD studies.
• PRé (the Netherlands) as one provider of LCA software used in higher education.
• Université de Sherbrooke (Canada), where the Interdisciplinary Research Laboratory in Life Cycle Assessment and Circular Economy (LIRIDE) integrated the concept of sustainable development and life cycle thinking into the bachelor, master and PhD programs (various disciplines of engineering).
• Solent University, Southampton (UK) where LCA is taught as an integrated topic across the engineering bachelor programmes and several PhD studies.
• The Zurich University of Applied Sciences (ZHAW), where life cycle thinking and the application of LCA results are integrated in various bachelor and master programmes (engineering and life sciences) and in-depth LCA competences are taught in a specific LCA minor for environmental engineers (Bachelor) and in advanced LCA courses in the masters programme on natural resource sciences.

The first expert panel provided an opportunity to discuss learning outcomes of different study programmes with incorporated LCA topics and key barriers of teaching and education of LCA. The second panel further developed a common understanding of learning outcomes, learning outcomes and competency levels achieved for different LCA courses. In the third panel, learning and teaching contents, materials, activities, and approaches were identified and categorized.

Given the large number of academic institutions teaching LCA, the expert panels which were carried out to complement the literature review provided a highly valuable yet limited insight into how LCA is taught around the world. In going forward, it is planned to carry out a comprehensive global survey to enhance the representativeness of the findings of the panels outlined in this paper as highlighted in our final chapter.

### Learning competences

The pedagogical methods and metrics behind the development of the framework on teaching LCA in higher education are described in this section.

An important aspect identified already in the first expert panel meeting was the need to adjust the content and level of complexity to the audience, considering the overall workload and specific LCA learning outcomes. To qualify the comparative time spent studying LCA topics, the European Credit Transfer and Accumulation System (ECTS) was used as reference, which presents a system for the transfer and comparability of accredited learning (EC 2015). Credits are awarded for completed learning, where one academic year normally corresponds to 60 ECTS-credits (equivalent to approximately 1500–1800 h of study, irrespective of qualification or standard). The system provisions for transferability between countries, and provides equivalence with country and university systems outside the European Higher Education Area, where a multitude of crediting approaches exist as exemplified in Table 1.

**Table 1:** Examples of credit equivalents to ECTS for one academic year (data taken from TCD 2020)

All ECTS-based institutions in Europe	60 ECTS
All UK-based institutions	30 credit
Chinese University of Hong Kong	48 credit
National University of Singapore	48 MCS
Peking University, China	30 credit
University of Melbourne, Australia	100 points
University of Southern California, USA	32 units

We applied the ECTS system to provide a gauge against which the comparative time spent studying LCA topics can be measured. The more time spent, the greater the credit allocation and the deeper the LCA teaching can be. The more time spent on study, broadly speaking, the higher the cognitive level at which learning can be achieved.

In line with cognitive levels, Bloom’s taxonomy is a set of hierarchical models used to classify educational learning outcomes into categories of complexity and specificity (Bloom et al. 1956). To differentiate cognitive skill levels within LCA teaching, Bloom’s taxonomy has been used. Originally presented to help develop rubrics (criteria for grading) and measure learning, the taxonomy encompasses six categories within the cognitive domain: knowledge, comprehension, application, analysis, synthesis, evaluation (Bloom et al. 1956). Bloom’s taxonomy is widely used for analysing and classifying cognitive skills in higher education and has been used in the LCA teaching context before (e.g. Favi et al. 2019; Olsen et al. 2018; Roure et al. 2018).

Bloom’s taxonomy has been modified by various authors since its original conception, with variations, modifications or additions (e.g. Anderson et al. 2001; Lytras and Pouloudi 2006). Most widely applied is the addition of a further category of ‘creation’ to the taxonomy, representing learner’s ability to create new knowledge at the point of achieving sophisticated understanding of a subject (Anderson et al. 2001). Table 2 lists and defines the categories of the revised taxonomy.

**Table 2:** Blooms' revised taxonomy (definitions taken from Krathwohl 2002)

Remember	Retrieving relevant knowledge from long-term memory
Understand	Determining the meaning of instructional messages, including oral, written, and graphic communication
Apply	Carrying out or using a procedure in a given situation
Analyze	Breaking material into its constituents parts and detecting how the parts relate to one another to an overall structure or purpose
Evaluate	Making judgements based on criterial or standards
Create	Putting elements together to form a novel, coherent whole or make an original product

A final important aspect to address are the learning outcomes. They are defined as the expected goals of a course, lesson or activity in terms of demonstrable skills or knowledge, which the students acquire and as a result of the delivery of taught content or teaching activities. It is useful to also consider these skills in relation to Bloom's taxonomy to provide appropriately levelled outcomes (Anderson et al. 2001). The terms learning outcomes and learning objectives are often used interchangeably. This paper uses the term learning outcomes to describe the intended overarching achievements of students in contrast to instructional and course-specific learning objectives (cp. e.g. Allan 1996, Harden 2002).

### Framework development

To develop a framework on teaching LCA in higher education, the results of the stakeholder workshops were structured in several categories: the degree of LCA integration into study programs measured in ECTS and workload, associated cognitive domain categories according to Bloom’s taxonomy, LCA learning outcomes, and envisioned professional LCA competency. These categories are used to define distinctive LCA learning and competency levels. To further develop the framework, common LCA teaching content and approaches are described, analyzed and categorized within the given levels.

## Results and discussion

Within this chapter, the outcome of the scientific literature review on LCA teaching (Section 3.1) and the results of expert panel discussion using the nominal group technique are utilized to conceptualize LCA learning and competency levels (3.2) and link these to their respective LCA teaching approaches and content with related workloads (3.3). If not stated otherwise, the results of Sections 3.2 and 3.3 are direct results of the expert panel discussions.

### LCA teaching experiences in the literature

Twenty-eight studies were identified in the literature and summarized in Table 3. The review aimed to identify published experience on teaching LCA in higher education. As highlighted by Burnley et al. (2019), LCA is taught in a significant number of higher education institutes across the world, but many do not publish papers on their teaching experience. Instead, this review reveals the current state of scientific discourse on teaching LCA and provides a basis for expert panel based assessment in later sections of this paper.

**Table 3:** Reviews of LCA teaching experiences, as identified in the literature

Paper	Scope		Focus				Presents course content	Use of LCA software				Number of hours and/or credits	Student background			Target audience		
	Course experience	Generic course proposal	Teaching method	LCA com-petences	Learning objectives	Literature review		Spread-sheet	Stream-lined software	Full LCA software	Not specified		Engineering and technical sciences	Business and social sciences	Not specified	Bachelor	Master	Doctoral
Balan; Manickam 2013	X			X			No				X	Not specified	X			X		
Burnley et al. 2019	X			X			Yes	X	X			Not specified	X	X			X	
Cosme et al. 2019	X		X	X	X		Yes			X		10 ECTS / 280 h	X				X	
Crossin et al. 2011	X		X				No			X		Not specified	X			X		
De Souza Xavier et al. 2014	X		X				No			X		Not specified	X			X	X	
Evans et al. 2008	X		X				No				X	Not specified	X			X		
Gilmore 2016	X				X		Yes			X		16 h	X			X		
Harding 2004	X		X				No		X			7 h	X			X		
Laurent et al. 2015	X				X		No				X	Not specified	X	X		X	X	X
Lockrey; Johnson 2013	X		X				No		X			24 h	X			X		
Loste et al. 2020	X		X				No				X	Not specified	X	X	X	X	X	X
Mälkki et al. 2016	X		X				No				X	Not specified	X			X	X	X
Mälkki; Alanne 2017		X				X	No				X	Not specified	X			X	X	X
Margallo et al. 2019	X		X				No				X	3 ECTS / 75 h	X				X	
Masanet et al. 2014	X		X				Yes				X	9 weeks MOOC	X	X		X	X	X
Meo and Brandt 2014	X		X				No		X			Not specified	X			X		
Mulder-Nijkamp et al. 2018	X		X		X		Yes		X			15 ECTS	X			X		
Olsen et al. 2018	X			X	X		Yes				X	From 5 to 50 ECTS	X			X	X	X
Olsen 2010	X				X		Yes				X	10 ECTS	X			X	X	X
Oude Luttikhuis et al. 2015	X			X			Yes			X		Not specified	X			X	X	
Perini et al. 2018	X		X				No				X	Not specified	X				X	
Piekarski et al. 2019	X		X				Yes			X		Not specified	X			X		
Roure et al. 2018	X			X			Yes		X	X		Not specified	X			X		
Sahakian and Seyfang 2018		X		X			No				X	2/3 h per week over one semester	X	X		X	X	
Sriraman et al. 2017a, b	X		X		X		No				X	Not specified	X	X			X	
Tasdemir Gazo 2020	X		X		X		Yes				X	16-weeks-long course	X			X		
Vallero Brasser 2008	X		X				No			X		Not specified	X			X		
Weber et al. 2014	X			X			Yes				X	Four weeks	X			X		

Through this literature review, it was confirmed that several universities implemented courses on LCA and environmental assessment tools in the past years. Most of the papers (26 out of 28) report an experience with an LCA course held at a specific university or different universities in the same country (identified as “course experience” in Table 3). For instance, Burnley et al. (2019) present a software tool developed at Cranfield University to allow part-time distance learning students to gain an understanding and experience of LCA. Olsen et al. (2018) and Cosme et al. (2019) present the LCA course experience at the Technical University of Denmark, including how students and companies are engaged in the course allowing a win–win situation to all stakeholders. De Souza et al. (2014) summarize experiences on graduate education in Brazil considering inputs from five different universities, and identified among others, common challenges among the partners as lack of national inventories to model LCA studies. In turn, Mälkki and Alanne (2017) aimed at understanding how LCA can be useful in renewable and sustainable energy education; a literature review was the starting point for the authors, and among other things, they concluded that LCA should be integrated into the learning outcomes of energy degree programmes. This last study was classified as a “generic course proposal”, although the authors do not suggest a detailed curriculum in the paper. As such, the different studies reflect different experiences from educators, but there is therefore no discussion nor comparisons between these different experiences.

In geographical terms, a predominant share of the studies (eleven) are based on experiences in European countries. The second region is North America with eight studies (seven from the United States and one from Canada), followed by Oceania (Australia) with three studies, South America (Brazil) with two studies and Asia (Malaysia) with one study. Three studies included several countries. Overall, this geographical distribution reflects the continental and regional evolution of LCA for the past 30 years, e.g. its use in industry (Stewart et al. 2018) or its spreading via LCA networks (Bjørn et al. 2013), where European and North American regions have seen the largest developments, while other regions like Asia or Africa lag behind with respect to LCA uptake.

Among the papers, ten included related approaches besides LCA, e.g. ecodesign, green chemistry, sustainable development, energy efficiency and circular economy (Loste et al. 2020; Oude Luttikhuis et al. 2015; Roure et al. 2018; Sahakian and Seyfang 2018). Although there is a consensus on the importance of considering sustainability in the academic curriculum of all areas, the majority of LCA courses identified (21) is applied to graduate or undergraduate students following an engineering or technology path. This may be explained by the traditional main use of LCA as a micro-level decision-support tool in industry for product or technology development, although it started to diversify in recent years with broader, large-scale assessments of organizations, sectors or countries (EC 2020; Laurent and Owsianiak 2017).

Different authors reported the relevance of project-based learning (e.g. Lockrey and Bissett Johnson 2013; Margallo et al. 2019; Piekarski et al. 2019; Sriraman et al. 2017a, b), most of the time through a life cycle assessment project developed by the students and using one or different LCA commercial software. Some studies presented experiences with cases developed in partnership with industrial partners (Cosme et al. 2019; Piekarski et al. 2019). As emphasized by Sriraman et al. (2017a, b), an appropriate pedagogy is essential to activate student engagement in the learning process and facilitate a deeper learning.

Regarding the structure of the courses, only a few studies presented the content of the lectures in details, and/or the learning outcomes (e.g. Cosme et al. 2019; Gilmore 2016; Margallo et al. 2019; Olsen 2010; Roure et al. 2018). Gilmore (2016) highlights the importance to adjust the content and level of complexity to the audience and the background of the students, but there is an evident absence of generic LCA course framework based on specific learning outcomes and teaching methods.

### LCA learning and competency framework

**Fig. 1:**
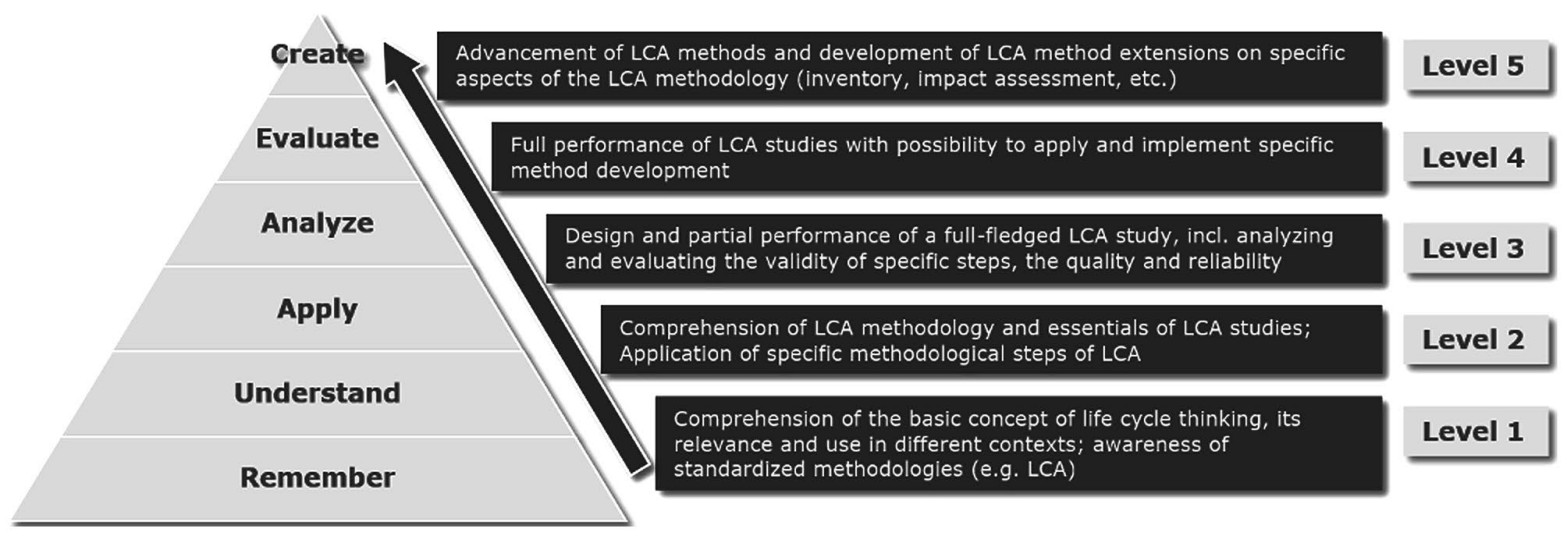
LCA learning outcomes and competency levels proposed within the frame of Bloom’s revised taxonomy

Figure 1 and Table 4 summarize the outcome of our expert panel research with regard to LCA learning and competency levels. Depending on the degree of study program integration and the respective workload in ECTS and hours, LCA teaching covers smaller or larger parts of Bloom’s taxonomy and pursues different learning outcomes which eventually result in nuanced expectations regarding the students’ professional life cycle literacy.

**Table 4:** LCA learning and competency framework for higher education (each level consists of one or more courses and courses between levels can add up)

LCA learning and competency level	level 1	level 2	level 3	level 4	level 5
study program integration	single lecture or module (part of a course centred on broader or other topics)	full module or individual course	minor specialization (several courses / modules)	major specialization (several courses / modules)	research specialization
ECTS	0—1	2 to 5	6 to 12	> 12	not defined
workload (hours) - total (in class | students' preparation, group work etc.)	up to 30 (15|15)	up to 150 (60|90)	up to 360 (120|240)	more than 360 (120|240)	not defined
Future/envisioned professional profiles of students	engineers and managers who can relate to LCA utilisation, commission LCA studies and take decisions/actions from LCA results	engineers and managers who can relate to LCA utilisation, commission LCA studies and take decisions/actions from LCA results	LCA practitioners or sustainability managers able to conduct and interpret LCA studies	LCA practitioners or sustainability managers able to conduct and interpret LCA studies, and possibly develop internal LCA-based tools catered to his/her organisation needs	LCA practitioners (incl. external reviewers), sustainability managers, researchers in academia
Learning outcomes	comprehension of the basic concept of life cycle thinking, its relevance and use in different contexts; awareness of standardized methodologies (e.g. LCA)	comprehension of LCA methodology and essentials of LCA studies; Application of specific methodological steps of LCA	design and partial performance of a full-fledged LCA study, incl. analyzing and evaluating the validity of specific steps, the quality and reliability	full performance of LCA studies with possibility to apply and implement specific method development	advancement of LCA methods and development of LCA method extensions on specific aspects of the LCA methodology (inventory, impact assessment, etc.)

Considering the modular structure of most higher education courses in LCA, we consider typical ECTS awards and Bloom’s taxonomy to derive four broad conceptual levels of learning outcomes and cognitive competency. For higher LCA competency levels, higher levels according to Bloom are involved: From the ability to gain or apply knowledge like the basic concept of life cycle thinking, to the ability to critically analyse and evaluate the quality of an own LCA study and finally being able to combine LCA results with insights from other disciplines to create new methods (see Fig. 1). These levels are intended to reflect the time spent by students studying LCA, reflected by the weight of ECTS credits available for part of a course, entire courses, modules or minor and major or complete programs. A fifth learning and competency level is additionally considered to capture research work undertaken by postgraduate research students, particularly in thesis work (MSc or PhD students). This reflects the suggestion of various revisions of Bloom’s taxonomy to add ‘creation’ as further category to the cognitive domain, which is not conceptual knowledge per se, but rather based on the understanding and skills gained through the preceding cognitive categories (Anderson et al. 2001). This fifth level is very flexible depending on the course specific focus and is therefore not detailed in further sections.

In Table 4 we suggest generic learning outcomes for LCA taught content corresponding to the five levels defined above. Through definition of topics, time, and cognitive categories for courses, instructors are able to more clearly define consistent learning outcomes. We recognize that instructors or curricula may have specific requirements or demands and therefore we suggest that these outcomes be applied and adapted as required. Based on the learning outcomes we come up with an envisioned professional LCA competency as the final outcome for students with regards to their future role as sustainability professionals. After their studies, some students will just be aware of the life cycle concept, they might commission LCA studies for decision-making in the future (level 1&2), some will be users of LCA information and contribute to their generation (level 3), while others will be able to conduct full-scale LCA studies (level 4), either in an accompanied or independent manner. As such, the level of LCA competency that will be required from students varies to a high degree.

### Application of the framework in higher education

The substantial differences in learning outcomes and envisioned professional LCA competencies do not only require distinct workloads and tailored study program integration, but also result in a large spectrum of LCA teaching activities, materials, and content. Therefore, this section showcases the use of the framework in higher education. Based on the expert panel research, we decided to focus on three broad types of LCA teaching: lecturing and similar activities, case studies and project work, and LCA software and database use. The latter is mostly part of case studies and project work and of particular relevance for LCA teaching in higher education.

The three types resemble Atkins and Brown’s continuum of teaching methods (2002) where lectures represent one end of the continuum with a high degree of engagement and control by the lecturer. The degree of student participation and independence then constantly increases via small group teaching, research supervision and lab work to the level of individual work (e.g. PhD studies) at the other end of the continuum. Accordingly, the addressed cognitive domain categories of Bloom’s taxonomy change from one type to the next.

The following sections further elaborate the three types (Sections 3.3.1–3.3.3), while typical teaching contents are addressed in Section 3.3.4. Unless stated otherwise, the results are the direct outcome of the nominal group technique.

#### Lecturing and teaching material

Lecturing is a typical means of communicating basic LCA content to students with lecturers presenting LCA topics, instructing small exercises and encouraging discussions. Given that lectures are the oldest and most common means of university teaching it does not require further explanation here. More student-centred learning activities are gaining importance in LCA teaching, e.g. e-learning or flipped classroom concepts. The latter suggests moving lecture material out of the classroom (e.g. via recordings to watch by the students before coming to the classroom) and using the classroom time for active learning, e.g. exercises and discussion (Lage et al. 2000). Teaching approaches are expected to change even faster due to the COVID-19 pandemic, which has already evoked a worldwide shift from classroom teaching to online teaching (cp. e.g. Bao 2020).

LCA lecturing is supported by textbooks, published guidelines, and online resources. Table 5 lists such LCA teaching documents, indicates the type of each document and its relation to the teaching and competency levels specified in Table 4. The specific role of standards and guidelines in teaching is to support and complement textbooks and other course material. A review of English textbooks is provided by Laurent et al. (2020).

**Table 5:** LCA sources used as teaching material (non-exhaustive list)

Type of document	Reference (year)	Related teaching and competency levels (tentative)
Textbooks	Bauman and Tillman (2004)	2–5
	Curran (2012)	2–5
	Finkbeiner (2016)	3–5 (focus on special LCA types)
	Frischknecht (2020)	2–5 (only in German)
	Fullana and Puig (1994)	2–5 (only in Spanish)
	Grisel and Osset (2004)	2–5 (only in French
	Guinée (2002)	2–5
	Hauschild and Huijbregts (2015)	3–5 (focus on LCIA)
	Hauschild et al. (2018)	1–5
	Heijungs and Suh (2002)	3–5 (focus on math. foundation)
	Jolliet et al. (2015)	2–5 (also in French)
	Klöpffer and Grahl (2014)	2–5 (also in German)
	Schenk and White (2014)	2–5
Online textbooks	Matthews et al. (2020)	2–5
	Sonnemann and Margni (2015)	3–5 (focus on LCM)
Online teaching material	ILCA (2020)	3–5
	UNEP (2020b)	1–4
	Uni Freiburg (2020)	1–4
Standards and guidelines (to support and complement textbooks and other course material)	Benoit and Mazijn (2009)—Social LCA (UNEP/SETAC)	3–5
	EC JRC (2010)—ILCD handbook	3–5
	EC JRC (2012)—PEF Guide	3–5
	ISO 14025 (2006)	2–5
	ISO 14,040 and ISO 14044 (2006)	1–5
	ISO 14046 (2014)	2–5
	ISO 14067 (2018)	2–5
	Sonnemann and Vigon (2011)—LCA DBs (UNEP/SETAC)	3–5
	Swarr et al. (2011)—LCC (SETAC)	3–5
	Valdivia et al. (2012)—LCSA (UNEP/SETAC)	2–5

#### Case studies and project work

Case studies and project work are very common and important elements of teaching LCA and appear at all levels of the teaching and competency level framework (Table 4). While case studies are often used for illustration and interpretation purposes at levels 1 and 2, the conduct of LCA case studies and projects, including data collection and other real-life challenges of LCA, takes place at levels 3 and 4.

On the first levels of the framework two main approaches can be distinguished:

**•** Case studies may be embedded into teaching LCA basics to support the understanding of main steps and procedures. For instance, after the functional unit and reference flow terminology has been introduced in a lecture (e.g. at level 2), groups of students reflect on this by making use of a given case study. Thereafter, the same order is repeated for product system, system boundaries, and so forth. Some textbooks (e.g. Klöpffer and Grahl 2014) support this “red thread” approach by integrating a continuous case study into each book chapter.
• Case studies are used subsequently to introductory lectures. Groups of students are for example requested to read and work on a given case study or elaborate a highly simplified case.

Typically, the case studies in levels 1 and 2 would relate to pre-made cases and not involve external collaboration. In some cases, software is used to support the case study work on these first levels (see also following section).

On higher levels of the competency framework (i.e. levels 3 and 4), the complexity and magnitude of case study and project works increase accordingly. Instead of just reading and interpreting elements of case studies or doing guided exercises on simplified case study material, students at levels 3 and above start to interpret and compare “full” LCA case studies and can also plan and execute full-fledged LCA studies by themselves including some parts of data collection and system modeling in LCA software. As a step further, student groups can conduct these LCA studies on real-life cases in collaboration with external partners (e.g. companies, municipalities), where they can experience challenges (e.g. data collection) and benefits (e.g. interactions and communication with engaged stakeholders) of real-life application of LCA.

These findings of the expert group panels suggest that group work and project work approaches, which stimulate active engagement by the students, are important to achieve desired LCA learning outcomes. This mirrors the outcome of the literature review in Section 3.1, where several authors (e.g. Margallo et al. 2019; Sriraman et al. 2017a, b) emphasize the importance of project- and problem-based learning approaches and others provide examples of real-life company cases being the core element of their teaching concepts (see Cosme et al. 2019; Lockrey and Bissett Johnson 2013; Piekarski et al. 2019).

#### Spreadsheet, software and database use

IT applications form an important part of LCA teaching and are often used to handle the quantity of data needed for Life Cycle Inventory (LCI) analysis and Life Cycle Impact Assessment (LCIA). While IT support can increase the understanding of LCA applications and better prepare the students for potential future careers as LCA practitioners, it also bears the risks of replacing or complicating the comprehension and critical questioning of LCA methodology. Defining the right dose of software use is hence an important part of LCA teaching. Besides the use of specialized LCA software and databases, spreadsheet exercises are a common part of LCA teaching. Spreadsheet exercises or by hand calculations enable first small LCA computations and help to illustrate LCA methods, which otherwise run automatically and therefore hidden in specialized software and database solutions. LCA teachers are hence encouraged to use LCA software to ensure that the mechanics behind LCA computations are well understood by the students, e.g. by small exercises with spreadsheets to mimic the computations of the LCA software at a lower scale (see Cosme et al. 2019 for an example).

Four types of LCA software for teaching purposes can be distinguished. Spreadsheet exercises (type 1) allow simple case studies or LCA calculations by displaying LCA results from simple LCI data multiplied by pre-calculated emission factors. Streamlined software applications (type 2) usually focus on one application (such as eco-design or footprinting), limited boundaries (e.g., cradle-to-gate) or limited sectors (e.g., buildings). Professional LCA software packages (type 3, e.g., SimaPro (PRé Consultants 2020), openLCA (GreenDelta 2020), GaBi (Sphera 2020), or Umberto (ifu Hamburg 2020) enable modelling of the life cycle of any products and calculate the associated environmental impacts based on several possible LCI databases for the background system and on several already implemented LCIA methods. Advanced use of LCA software through coding or programming (type 4) such as (i) stand-alone programming framework (e.g., Brightway2 (Mutel 2017)) and (ii) COM-interface to automatically run LCA software from a third-party application of programming language.

As presented in Table 3, types 1,2 and 3 software are used as a support for LCA classes and for doing exercises or conducting student projects. Type 4 are mostly used at PhD levels. However, the development of programming classes in curricula might foster the use of type 4 software in LCA classes at competency levels 3 and 4.

**Fig. 2:**
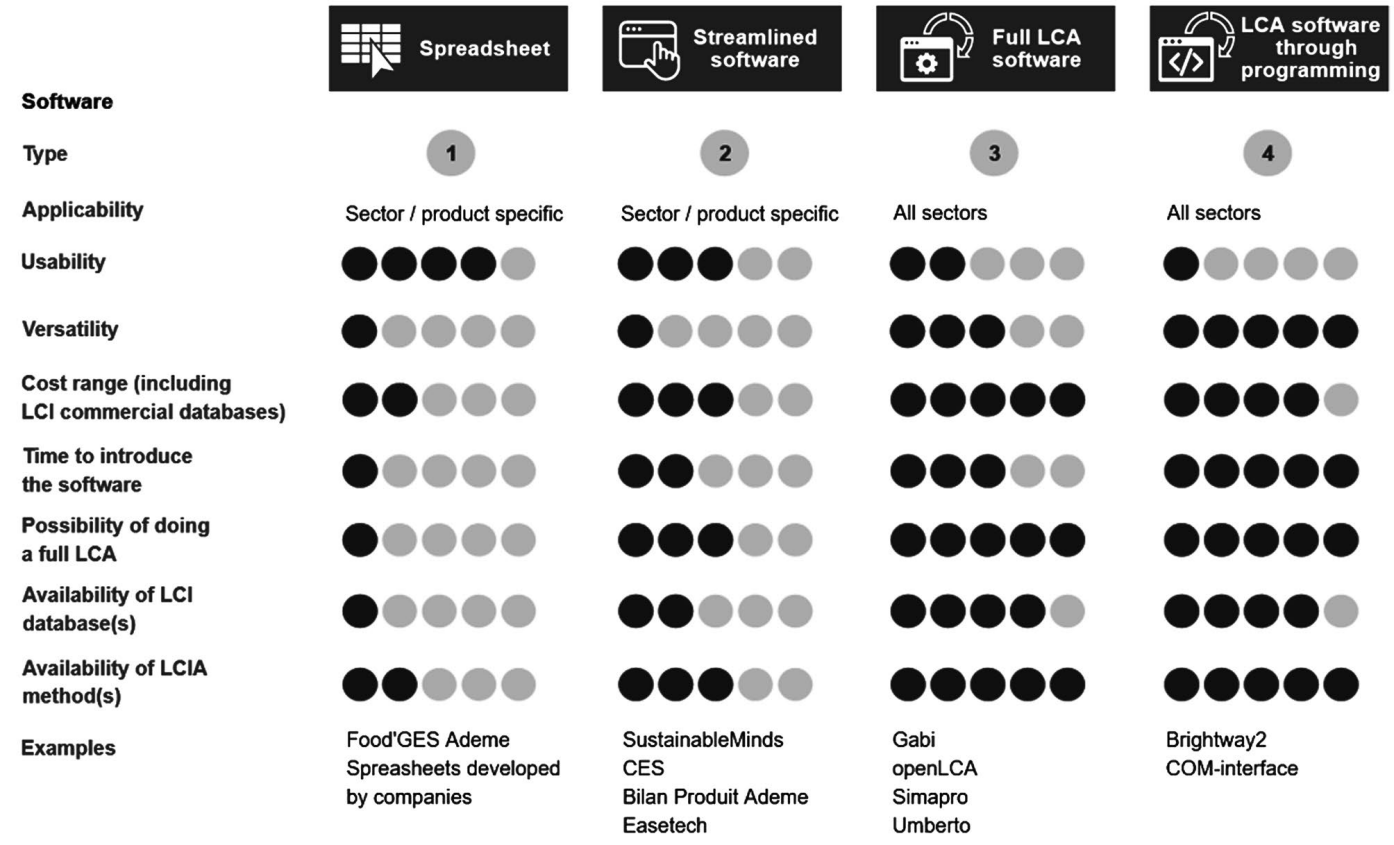
Comparison of four types of software to teach LCA (Levels: B = Bachelor, M = Master, D = Doctoral)

When designing an LCA class and selecting an LCA software, several aspects need to be considered, including financial feasibility (“cost range”), the product or activity to be studied (“applicability”), the ease-of-use during the learning process (“usability”), the different functionalities available in the software (“versatility”), the time dedicated to learn and use the software, the need to conduct a partial or full LCA, the need to use several LCI databases and/or several LCIA methods, the level of the class concerning LCA (according to Table 4), and the associated competencies to be learned. Figure 2 represents and compares most of these aspects for the types of software mentioned above.

Access to LCI databases is also an important aspect when using LCA software for teaching. There are several educational and free of charge LCI database packages available. However, advanced LCI modelling as well as conducting real-life case studies require access to comprehensive databases that are often commercial (e.g. ecoinvent and GaBi). At advanced LCA competency levels, students might work on LCI datasets from such databases, e.g. to perform adaptations to specific regional settings or to conduct sensitivity analyses.

#### LCA teaching content

As LCA is an interdisciplinary approach, its concepts and topics are taught at various levels and across a range of subjects from engineering to management, to dedicated industrial ecology courses. Subsequently, the time spent, teaching content and methods covered, and depth of learning varies widely from single classes and modules to entire courses, at both under- and postgraduate levels. Also, the number of students/participants and the available teaching staff resources affect teaching content and related activities.

LCA teaching content varies from topics like introduction to life cycle thinking via life cycle inventory modeling and environmental impact assessment methods to the mathematical foundations of LCA. The typical workload for students goes from awareness-raising and basic knowledge acquisition of up to 30 h as part of courses, through entire courses and modules of up to 150 h, to specialized minor and major with more than 360 h in studies of different disciplines. Not all this workload is necessarily dedicated to lecturing; in particular in higher levels of competencies, case studies and group work are a major element of the course (see Section 3.3.2), meaning that lecturing may only represent a third or half of the total workload (see Table 4).

The teaching starts in general by introducing the wider context of LCA (e.g. sustainable development, resource efficiency, circular economy, environmental management and eco-design), the basic understanding of environmental impacts (e.g. climate change and acidification) and the concept of life cycle thinking as a way of providing a holistic understanding of value chains and product systems. Often jointly with this concept, the trade-offs or burden shifting across life cycles and impacts is explained. This broad introduction is followed by the description of the purposes of LCA jointly with illustrations of the lectures by use cases corresponding to the students’ area of study. As a next step, generally the ISO 14,040/44 framework including the LCA phases and its terminology (functional unit, system boundaries etc.) are clarified. These teaching contents are considered necessary to achieve the learning outcomes that students understand life cycle thinking and its utilization and relevance.

Students who are supposed to use LCA methodology and understand the basics of LCA studies need to learn more details on goal and scope definition, inventory modeling, impact assessment methods and the interpretation of results. They should also be aware of special types of LCA. That means carbon and water footprinting, life cycle sustainability assessment, including social LCA and life cycle costing, organisational LCA and/ or Input–Output and hybrid LCA. Moreover, multiple applications of LCA in specific sectors of relevance for the students (such as e.g. building, materials or energy) are explained more deeply. Finally, a basic understanding of how LCA is implemented in industry for innovation and communication through the concept of Life Cycle Management is often part of this level.

At higher learning and competency levels, classes often also include sensitivity and uncertainty analyses, the mathematical foundation of LCA and stimulate system thinking with the combination of LCA with other system-analytical tools like material flow analysis (MFA) and environmental risk assessment (ERA). Moreover, the more hours the topics mentioned above are taught, the higher the level is, with the exception of level 5 where the students mostly learn in an autodidactic way within a project coached by a professor or researcher, but not through classroom formats.

To systematize this diversity of LCA teaching content, the expert panels conflated the LCA learning and competency levels from Table 4, their workshops’ collection of teaching topics, and the three types of LCA teaching approaches from previous sections. Subsequently, the expert panels assigned typical workloads for students for each topic at each level. Table 6 consolidates these findings and presents an overview of typical teaching content at the different levels. For instance, the typical workload for introducing the wider context of LCA (e.g. its role within eco-design etc.) is about 2 h on level 1, but up to 8 h on higher levels (see first line in the classroom format category). While level 1 aims to provide a basic understanding of environmental impacts such as climate change within 4 h of workload, higher levels typically double this (see second line in the classroom category). In addition, levels 2, 3, and 4 enable more specific insights into environmental impact assessment methods with typical workload of 8, 16, and 32 h respectively (see row on LCIA in the lectures category). While Table 6 provides a good overview of typical topics and time requirements at each LCA competency level, it is not meant to be exhaustive. It furthermore does not detail the required abilities for each item. These abilities are reflected by Bloom’s cognitive categories that are juxtaposed to the LCA levels in Fig. 1. It is recommended to incorporate these categories into the planning and conduct of LCA courses for each given topic in Table 6.

**Table 6:** Recommended LCA teaching content in higher education based on learning and competency levels (workload on each level is independent, not cumulative; content in rows is not mutually exclusive)

	LCA learning and competency level	level 1	level 2	level 3	level 4	level 5
Type of teaching	Content, topics	anticipated workload for students (tentative hours, upper estimate)				
Lectures and similar formats	Wider context of LCA (e.g. SDGs, resource eff., circular ec., env. management, eco-design)	2	4	8	8	varies depending on individual research objectives of PhD or research-oriented master program/thesis
	Basic understanding of environmental impacts (e.g. climate change, acidification)	4	8	8	8	
	Life Cycle Thinking (holistic understanding of value chains / product systems)	4	6	6	6	
	Purposes and use cases of LCA	2	6	6	6	
	ISO 14,040/44 Framework (Life cycle phases and process according to ISO)	4	4	4	4	
	LCA Terminology (functional unit, system boundaries etc.)	4	8	8	8	
	Trade-offs or burden shifting across life cycles and impacts	2	6	12	12	
	Goal & Scope—definition		6	12	12	
	Goal & Scope—Decision context of LCA (attributional, consequential etc.)		4	8	12	
	LCI modeling and data: fore- and background		10	16	20	
	LCI modeling and data: multifunctional systems (system expansion etc.)		8	14	20	
	LCIA—Environmental impact assessment methods		8	16	32	
	Interpretation of results		8	18	24	
	Interpretation—sensitivity and uncertainty analysis			12	20	
	Special types of LCA (from CFP&WFP via LCSA to O-LCA&EEIO-LCA)		8	16	36	
	Life Cycle Management (use of LCA results for innovation & communicaton, etc.)		8	16	28	
	Applications of LCA (for emerging technologies, in specific sectors and in policy, etc.)		8	10	24	
	Mathematical foundation of LCA			10	20	
	Integration of LCA with theories (e.g. micro-economics, behavioral science) and system-analytical tools (MFA, RA, etc.)			10	20	
Case study and project work	Interpretation of a case study	4	8			
	Reproduction of a case study		16			
	Review / meta-analysis of LCA cases				40	
	Data collection and preparation			20	60	
	Conduct of a simple case study		30	90		
	Conduct of comprehensive case studies			90	160	
Software and database use	Simple LCA software (type 1 and/or 2) examples	4				
	Advanced LCA software (type 2, 3 and/or 4) use		12	40	80	
	Excercises in and comparison of LCI databases and/or LCIA methods			10	20	
	Excercises in and comparison of several LCA software				40	

Table 6 illustrates the increasing diversity of teaching approaches with a high degree of student participation and independence as the learning and competency levels increase (for competency levels, see Chapter 3.2). Case study and project work as well as software and database use form larger portions of the overall workload, increasing from about a quarter of the total workload on levels 1 and 2 to more than half of the workload at level 4.

## Conclusions and perspectives

The framework developed in this paper describes five LCA learning and competency levels in higher education institutions. It aligns the topics that should be addressed with the total workload needed to achieve the different competency levels. The learning outcomes of the LCA competency levels are linked with Bloom’s taxonomy, with the higher levels covering a larger share of Bloom’s cognitive categories, also reflecting the importance of critical and system thinking within LCA. The framework is proposed as a practical tool for those involved in the delivery of LCA education in higher education. On the one hand, it provides guidance on the workload, which needs to be invested in order to fulfil the learning outcomes for different LCA competency levels. On the other hand, it allows determining which approaches and content can be taught given a pre-set workload or number of available hours. Moreover, teaching documents that can be used as support material are cited.

This work does not include empirical research but represents the consolidated findings of LCA teachers and other stakeholders on the topic following the nominal group technique. The results represent an average of various study programs and disciplinary backgrounds and thus cannot reflect each individual study program in all aspects. The results are based on the view of various experts mainly from Europe but also from North America who have experience in teaching LCA. Since they are not adequately representing the perspective of Africa, Asia and Latin America, the results are clearly not representative for the world, in particular not for emerging economies. Overall, the paper does contribute to a better understanding of teaching LCA in higher education by providing structured guidance and a framework on LCA learning and competency levels with related teaching approaches and content. It encourages and supports higher educational bodies and their staff to apply a minimum of ‘life cycle literacy’ to students across various disciplines by increasing the availability, visibility and quality of their teaching on life cycle thinking and LCA. The framework also reveals that educating competent and independent LCA experts requires comprehensive and interdisciplinary LCA teaching, which goes beyond simplified and mechanistic approaches and encourages critical systems thinking.

In that setting, we provide sets of recommendations for users of the framework:

• Curriculum developers can use the framework to determine the amount of credits that would need to be assigned per course to allow students to acquire the different competence levels.
• University professors who are interested in offering courses on the subject can get orientation and, based on Table 6, can choose the content that corresponds to the level of their students as well as the available time.
• Students can use the overview to compare their course descriptions and contents with the competency levels listed in Table 4 in order to self-assess their own capabilities and they can manage their expectations on which time investment will lead to what level of competency in LCA.
• LCA-software providers and LCI database providers can profit from the overview of skills listed for different competency levels to create suitable services for each level.
• Prospective employers who want to recruit a person with an LCA background can use the framework to better determine the actual qualifications that they expect from their candidates.

Future research might enhance the framework by triangulating its results further, e.g. through conducting empirical surveys to gather the knowledge and experience of LCA teachers in higher education and beyond globally, using a bottom-up approach instead of the top down approach applied in this paper. This could extend the insights into teaching LCA in higher education by providing a mapping of all programs and courses available worldwide. This could also pave the way for developing a repository or platform providing all LCA teaching resources available in the world.

The results of such a new research project would help students to find a qualification and education that best fits their prospective careers and they would show educational institutions and their staff which options for integrating LCA are available. In addition, the overview could provide contact details that would help students to orientate themselves and institutions to set up free online platforms to foster knowledge exchange on teaching practices.

Furthermore, the current focus of the framework on higher education could be broadened by including lifelong learning activities as envisioned for instance by the Life Cycle Assessment Certified Professional programs of the American Center for Life Cycle Assessment (ACLCA 2020). Lifelong learning is crucial since it assures that practitioners keep or increase their LCA competency level after their initial training and that they can continuously provide high quality LCA studies.

Finally, opportunities exist to use the framework to foster ‘life cycle literacy’ in a more systematic way by working not only with curriculum developers but also policy-makers, who want to promote a more sustainable economy based on scientifically sound information. These stakeholders may have interest in ensuring that ‘life cycle literacy’ is built, and thus could use the framework to find out what kind of knowledge is currently taught by universities in their region. They could then engage in collaborations with curriculum developers to foster the teaching on life cycle thinking and LCA in their higher education institutions. FSLCI and other organizations working on encouraging the use of life cycle information worldwide could become facilitators for promoting the growth or ‘life cycle literacy’ to those stakeholders based on the LCA learning and competency level framework for higher education presented in this paper.
